# Supplementary material for: Prognostic Role of Circulating Tumor Cells in Patients with Metastatic Castration-Resistant Prostate Cancer Receiving Cabazitaxel: A Prospective Biomarker Study
Source: Cancers (Basel). 2023 Sep 11;15(18):4511. doi: 10.3390/cancers15184511 (PMC10527446; doi:10.3390/cancers15184511)

Supplemental Table S1: Response according to CTC status at baseline

	CTCs - CS≥1* (n=57)			CTCs - CS≥5* (n=57)		
	(-) n=9	(+) n=48		(-) n=20	(+) n=37	
	N (%)	N (%)		N (%)	N (%)	
PR	2 (22.2)	1 (2.1)		2 (10.0)	1 (2.7)	
SD	3 (33.3)	10 (20.8)		6 (30.0)	7 (18.9)	
PD	4 (44.4)	37 (77.1)		12 (60.0)	29 (78.4)	
			p-value			*p-value*
PR+SD	5 (55.6)	11 (22.9)	**0.099	8 (40.0)	8 (21.6)	***0.141
PD	4 (44.4)	37 (77.1)		12 (60.0)	29 (78.4)	

*per 7.5ml of peripheral blood ** Fisher's exact test *** Pearson Chi-Square

Supplemental Table S2: PFS according to CTC status at baseline

	CTCs - CS≥1* (n=57)			CTCs - CS≥5* (n=57)		
	(-) n=9	(+) n=48		(-) n=20	(+) n=37	
**Kaplan-Meier**			*p-value*			*p-value*
Events	8	44	0.517 (Log rank test)	17	35	0.089 (Log rank test)
Median	4.3	4.1		5.4	3.7	
Min-Max	1.0 - 7.1	1.0 - 17.9		1.0 - 15.6	1.0 - 17.9	
95% CI	1.9 - 6.8	3.5 - 4.6		4.5 - 6.2	3.0 - 4.4	

*per 7.5ml of peripheral blood

Supplemental Table S3: PFS and OS according to CTC status at Disease Progression

a. PFS

	CTCs - CS≥1* (n=20)			CTCs - CS≥5* (n=20)		
	(-) n=3	(+) n=17		(-) n=7	(+) n=13	
**Kaplan-Meier**			*p-value*			*p-value*
Events	3	14		5	12	
Median	5.4	4.2	0.536 (Log rank test)	5.4	4.1	0.380 (Log rank test)
Min-Max	4.3 - 8.8	1.5 - 15.6		3.8 - 8.8	1.5 - 15.6	
95% CI	3.7 - 7.0	3.7 - 4.7		4.3 - 6.4	3.5 - 4.6	

b. OS

	CTCs - CS≥1* (n=20)			CTCs - CS≥5* (n=20)		
	(-) n=3	(+) n=17		(-) n=7	(+) n=13	
**Kaplan-Meier**			*p-value*			*p-value*
Events	2	5		2	5	
Median	Not est.	Not est.	0.881 (Log rank test)	**28.0	16.4	0.152 (Log rank test)
Min-Max	14.5 - 28.0	2.5 - 33.9		7.4 - 33.9	2.5 - 24.2	
95% CI	-	-		8.4 - 47.5	2.2 - 30.5	

*per 7.5ml of peripheral blood; **Estimated (not actuarial)

Supplemental Figure S1: PFS for CTC-positive and CTC-negative patients at baseline (cutoff: 5 CTCs/7.5ml of PB)

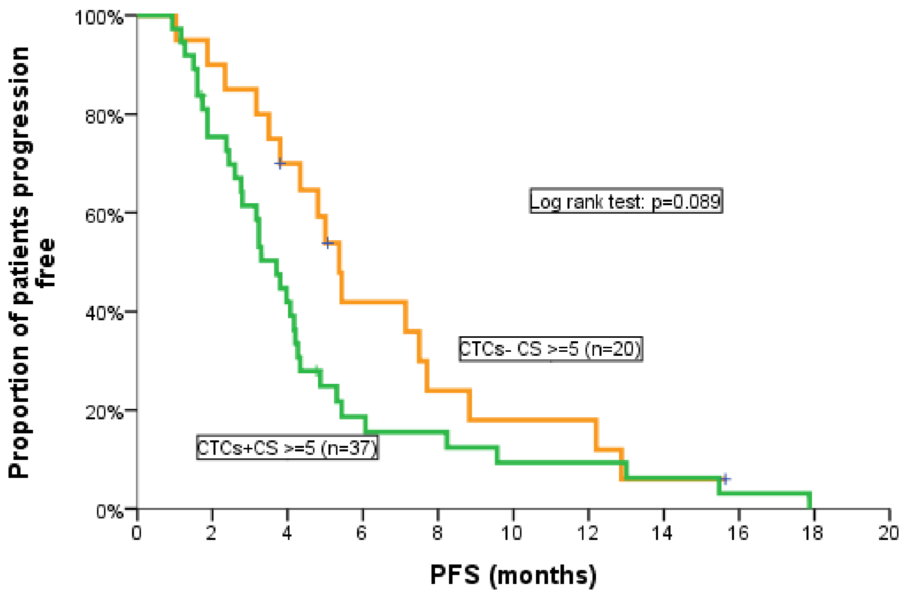

Supplement: Supplementary file 1 [file cancers-15-04511-s001.zip › cancers-2538267-supplementary.pdf]
